# *speckle-tracking*: a software suite for ptychographic X-ray speckle tracking^1^

**DOI:** 10.1107/S1600576720011991

**Published:** 2020-10-19

**Authors:** Andrew J. Morgan, Kevin T. Murray, Harry M. Quiney, Saša Bajt, Henry N. Chapman

**Affiliations:** a Center for Free-Electron Laser Science, DESY, Notkestrasse 85, 22607 Hamburg, Germany; b ARC Centre of Excellence in Advanced Molecular Imaging, School of Physics, University of Melbourne, Parkville, Victoria 3010, Australia; c DESY, Notkestrasse 85, 22607 Hamburg, Germany; d The Hamburg Centre for Ultrafast Imaging, Luruper Chaussee 149, 22761 Hamburg, Germany; e Department of Physics, Universität Hamburg, Luruper Chaussee 149, 22761 Hamburg, Germany

**Keywords:** software, wavefront metrology, speckle tracking, ptychography, X-ray projection imaging

## Abstract

In recent years, X-ray speckle-tracking techniques have emerged as viable tools for wavefront metrology and sample imaging applications. These methods are based on the measurement of near-field images. Thanks to their simple experimental setup, high angular sensitivity and compatibility with low-coherence sources, these methods have been actively developed for use with synchrotron and laboratory light sources. Not only do speckle-tracking techniques give the potential for high-resolution imaging, but they also provide rapid and robust characterization of aberrations of X-ray optical elements, focal spot profiles, and sample position and transmission properties. In order to realize these capabilities, software implementations are required that are equally rapid and robust. To address this need, a software suite has been developed for the ptychographic X-ray speckle-tracking technique, an X-ray speckle-based method suitable for highly divergent wavefields. The software suite is written in Python 3, with an OpenCL back end for GPU and multi-CPU core processing. It is accessible as a Python module, through the command line or through a graphical user interface, and is available as source code under Version 3 or later of the GNU General Public License.

## Abstract 2

The program *speckle-tracking* is described, an open-source software suite for performing wavefront metrology and sample imaging from projection in-line holograms of a sample.

## 1. Introduction   

The X-ray speckle-tracking (XST) technique was introduced by Bérujon *et al.* (2012 ▸) and a little later by Morgan *et al.* (2012 ▸) (no relation to the current author AJM) as a way of obtaining a quantitative measurement of the phase of a wavefield, as induced by a transmitting object, for example. The general wavefront-sensing principle of XST can be understood in terms of geometric optics, where it is assumed that light rays are directed normal to the isosurfaces of a wavefield’s phase. An analyser object is placed in that wavefield and used to map the directions of ray paths. The analyser is typically chosen to be a thin sample with a random phase/absorption profile that produces intensity variations (or ‘speckles’) some distance downstream, in the near field. These speckles are used as fiducial markers to determine the directions of the rays in the wavefield.

By measuring these speckle patterns at two detector distances, the lateral translation of speckles from one plane to the next allows the direction of a ray to be triangulated. This measurement strategy is termed the ‘absolute configuration’ of XST, in the sense that the phase profile of the wavefield is recovered, regardless of whether those phase distortions originate from any particular upstream optics or sample. If, rather than two detector distances, two images are recorded at a single detector distance, one with and one without the presence of an additional sample, then the speckle translations encode the phase distortions caused by transmission through that sample rather than the wavefield itself. This measurement strategy is termed the ‘differential configuration’ of XST, in the sense that the recovered phase profile is the difference between the phases of the wavefield before and after insertion of the sample.

The ray path directions obtained through the absolute-configuration XST method yield the 2D phase gradient of the wavefield. Integrating these 2D phase gradients then provides the phase profile of the wavefield. Coupled with the wavefield’s intensity profile, the wavefield can then be numerically propagated using wave optics from the measurement plane to any points of interest, such as the focal spot of a lens, the sample entrance surface or the lens pupil plane.

Since 2012, a number of XST-based methods have been developed, as reviewed by Zdora (2018 ▸). A more recent method is the ptychographic X-ray speckle-tracking (PXST) technique (Morgan, Quiney *et al.*, 2020 ▸). PXST was developed as a wavefront metrology technique that is optimized for the highly divergent wavefields produced by wedged multilayer Laue lenses (Morgan, Murray *et al.*, 2020 ▸). Note that PXST is not the only XST-based technique that can be used in the presence of highly divergent wavefields, nor does it provide some of the sample imaging modalities that can be obtained with alternative approaches.

In PXST, a dedicated speckle-producing analyser object is not used, and near-field speckle images are recorded at a fixed detector distance as the sample is scanned across the wavefield in the plane transverse to the optical axis. In the absolute configuration of Berujon’s original proposal, the location of each ‘speckle’ (here generated by the sample) in each image would be compared with those observed in a plane further upstream or downstream of the current detector plane. But this approach is impractical for highly divergent wavefields, since the footprint of the wavefield on the detector will be either much smaller or much greater than the detector’s width. In the differential configuration of XST, the wavefield’s ray paths could also have been determined by comparing the location of the observed speckles with those observed in an image of the same sample, but illuminated with an ideal wavefield, such that the ray path directions are known *a priori*. But again, such an approach is impractical in cases where the optics to produce such an idealized wavefield are not available. In PXST, the missing reference image is instead recovered from the data themselves, which is possible due to the high degree of redundancy in the data. This step – extracting the virtual reference image from the data – adds computational complexity to the wavefront reconstruction algorithm but simplifies the experimental procedure. It also allows for large magnification factors in cases where it would otherwise be impractical to measure the reference image, which in turn allows for very precise measurements of a wavefront’s phase and ray angles (to nano-radian accuracy).

In PXST, the sample acts like the wavefront analyser but it need not necessarily have the properties that produce a speckle-like pattern in the near field. Indeed, as seen in the examples shown in Figs. 3 and 4, the approach generally works as long as features in the pattern (in those cases a Siemens star object) can be identified in several images. The result of the technique is a map of the phase and amplitude of the stationary wavefront (the ‘illumination’) and a map of the near-field pattern of the sample as would be obtained in a perfect wavefield, with an arbitrarily large field of view (Morgan, Murray *et al.*, 2020 ▸). As such, by separating the effects on the measurement of a stationary wavefield and a translated object, the method is similar to near-field ptychography (Stockmar *et al.*, 2013 ▸).^2^ However, unlike that approach we rely upon ray optics rather than diffraction (Morgan, Quiney *et al.*, 2020 ▸).

**Figure 1:**
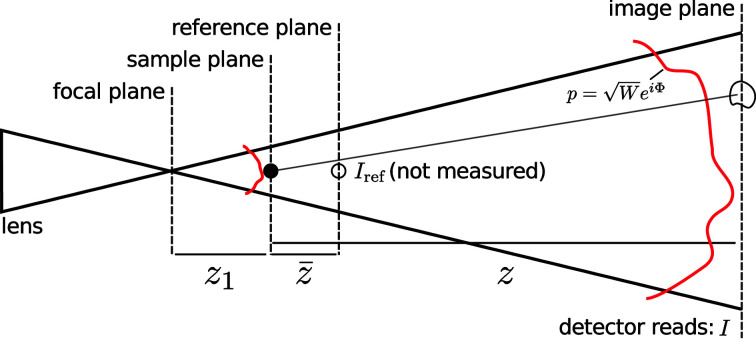
A schematic diagram for a projection imaging experiment. The illuminating beam propagates from left to right and the solid black lines indicate the boundaries of the illumination wavefront. The sample is depicted as a small black filled circle in the sample plane and as a black open circle in the reference and image planes. The red lines depict the illumination’s wavefront in the sample and image planes, which are not merely related by transverse magnification. The distorted shape of the circle in the image plane represents possible distortions of the speckle produced by the sample and the transverse phase gradients of the illumination.

In this article, we consider the general case of an imaging geometry corresponding to the projection imaging experiment as shown in Fig. 1 ▸. Here, due to the beam diverging from the focus of a lens, the detector records a magnified near-field projection image (or hologram) of the sample. The equation relating the *n*th recorded image (*I*_*n*_) to the (un-recorded) virtual reference image (*I*_ref_) in terms of the wavefront’s phase profile (Φ) is given by [equation (25) in the paper by Morgan, Quiney *et al.* (2020 ▸)]
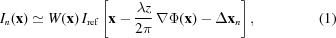
where **x** ≡ (*x*, *y*) is a vector in the plane transverse to the optical axis, 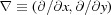 is the transverse gradient operator, the detector is a distance *z* downstream of the sample, the wavelength of the monochromatic wavefield is λ, Φ and *W* are the phase and intensity of the illumination’s wavefront, respectively, in the plane of the detector, and the sample displacement in the transverse plane corresponding to the *n*th image is Δ**x**_*n*_. The reference image corresponds to a magnified image of the sample one would have observed with plane wave illumination, covering the entire illuminated area of the sample throughout the scan, on a detector a distance 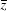 from the sample plane. In practice, for *W* ≃ 1 and within the limits of the approximation in equation (1), the reference image is simply a merging of each recorded image in the scan after correcting for the geometric distortions induced by the lens aberrations. The displacements of these overlaid images are given by the demagnified sample positions Δ**x**_*n*_/*M*, where *M* is the magnification factor (see below).

For an ideal lens system producing an unaberrated diverging wavefield, 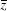 = 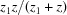 = *z*/*M*, where *z*_1_ is the focus-to-sample distance and *M* is the effective magnification factor. In Fig. 1 ▸ we show the relative positions of the focal plane, the sample plane, the reference image plane and the image plane (where the detector is located). The central goal of the *speckle-tracking* software suite introduced here is to solve the set of equations in equation (1) for Φ and *I*_ref_ in terms of the recorded images *I*_*n*_.

## 2. Main functions and overview   

Our intent is for the software described here to be accessible to imaging scientists and engineers who may not necessarily be experts in any particular field of imaging. In addition, we have designed the software suite so that it might be integrated into the standard processing pipelines at beamlines and laboratory sources around the world. To these ends, we have developed three modes of user interaction with the basic routines of the suite: (i) a Python module interface, so that users have access to each of the low-level functions of the suite and may modify or replace such functions for different applications; (ii) a command-line interface, to enable automated processing of data sets that may run in batch mode or remotely on a computing cluster; and (iii) a graphical user interface (GUI), designed for users who wish to explore their data sets interactively and troubleshoot for possible sources of error in the analysis. Python was chosen as the top-level language due to its ease of use, its open source licence and its wide adoption by the scientific community. The more intensive calculations are executed in OpenCL, which can be run on multi-CPU hardware as well as Intel, NVIDIA and AMD-based GPUs. OpenCL was chosen over alternatives such as CUDA, for example, which can only be executed on NVIDIA-based GPUs. The OpenCL kernels are compiled and interfaced with the Python routines at run time via the PyOpenCL API (Klöckner *et al.*, 2012 ▸). If the external dependencies of the suite are present, then the software does not require any installation or initial compilation. This further simplifies the setup process and possible integration with other processing pipelines.

Other groups have also distributed the source code for implementing a particular variant of the XST approach. For example, Paganin *et al.* (2018 ▸) have released the source code and experimental data for the ‘single-image geometric flow’ XST algorithm, available at https://github.com/labrieth/spytlab. Zdora *et al.* (2017 ▸) have also released the source code implementation for their *Unified Modulated Pattern Analysis* (*UMPA*), available at https://github.com/pierrethibault/UMPA.

**Figure 2:**
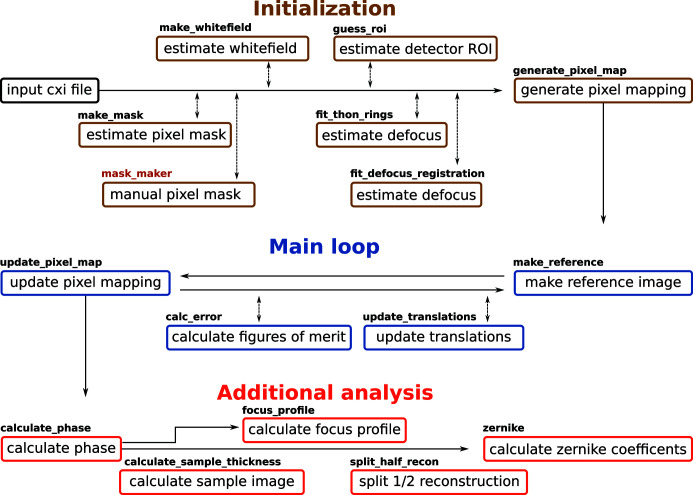
A flow diagram of the main operations in a PXST reconstruction. The name of the process for executing each step is displayed in bold above the description.

The order in which the main functions performed by the *speckle-tracking* software suite may be executed is illustrated in Fig. 2 ▸. Each of these functions can be called directly via the Python module, where the input data and algorithm parameters are supplied by standard Python objects. Corresponding to each of these functions is a command-line script of the same name. In each script, the input data are extracted from a CXI file (see Section 3 below), with optional parameters read from a configuration file. The command-line script then executes the corresponding Python function and returns the output into the same CXI file (unless otherwise configured). For each command-line script there is a corresponding GUI, where the algorithm parameters can be set, the command executed and the output visually examined. The display routines are based on the *pyqtgraph* project (http://www.pyqtgraph.org), which allows for fast and interactive visualization of large data sets.

**Figure 3:**
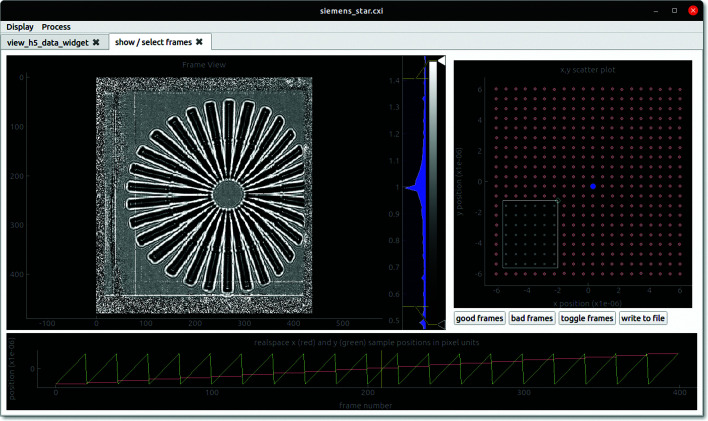
A screen shot of the frame_viewer GUI. The *x* and *y* coordinates of the sample translations (Δ**x**_*n*_) are displayed as both line plots (bottom panel) and a scatter plot (top right panel). The sample position corresponding to the current image is indicated by the vertical yellow line and the blue dot, respectively. The current image is displayed in the top left panel, which will be divided by the white-field image, if present, in order to increase the contrast. One can scroll through the images by dragging the yellow line. Rather than read the entire data set into system memory, each image is read from file in real time so that very large data sets can be accommodated. The white rectangle in the top right panel can be used to select several frames to exclude from further analysis. The good (red dots) and bad (grey dots) status of a frame can also be toggled with the left mouse button.

There is also a small set of additional GUIs (the first two are not shown in Fig. 2 ▸) that are not linked to a specific command-line script:

(i) hdf5_viewer displays the contents of the CXI file and allows for basic visualization of its constituent data sets.

(ii) frame_viewer allows viewing of individual images in correspondence with the transverse coordinates of the object. In this GUI one can manually exclude images from further analysis (see Fig. 3 ▸).

(iii) mask_maker allows the user to edit the mask file of the detector manually. The mask is displayed as an overlay on top of the raw data, which allows the user to spot bad pixels that may not have been picked up from the automatic processing (*e.g.* via the make_mask function).

Equation (1) describes the relationship between the reference image, *I*_ref_, and the *n*th recorded image, *I*_*n*_, as a geometric mapping defined in terms of the phase gradients ∇Φ and the sample position Δ**x**_*n*_. So equation (1) can be expressed compactly as *I_n_*(**x**) = *W*(**x**) *I*_ref_[**u**(**x**) − Δ**x**_*n*_], where **u** is that component of the geometric mapping that remains constant from image to image, 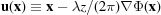. In the *speckle-tracking* suite, the reference image and the mapping function are solved for in an iterative update procedure that aims to minimize the target function [equation (26) in the paper by Morgan, Quiney *et al.* (2020 ▸)]
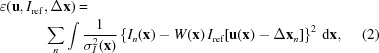
where 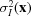 is the variance of the intensity signal at **x** over the *n* recorded images.

This iterative update procedure (described below) amounts to an alternative minimization of equation (2), so that ∊ is progressively minimized with respect to each of its arguments, in series, until convergence has been achieved within a given tolerance level. The minimizations with respect to **u** and Δ**x** are performed via a brute-force grid-search algorithm, while the update for *I*_ref_ can be calculated directly, since ∊ is a quadratic function of *I*_ref_. This approach, which is also termed coordinate descent, is a local optimization strategy and does not guarantee convergence from an arbitrary starting point (Bezdek & Hathaway, 2002 ▸).

### 2.1. Initialization   

Ideally, one would already have several maps and quantities before performing the wavefront reconstruction. These are (i) the pixel mask (*M*), which indicates detector pixels common to all images *I*_*n*_(**x**) to exclude from subsequent analysis (equal to 1 for good pixels and 0 for bad); (ii) the white-field image (*W*) equal to the image that would be recorded without any sample present; (iii) the sample defocus (*z*_1_); and (iv) the detector’s region of interest (ROI), which is a rectangular region of the detector used in the analysis. In sitations where the ROI is non-rectangular, for example where the lens pupil is circular or elliptical, the pixels outside of this region can be excluded via the pixel mask. In such a case, the only initialization required in the processing pipeline is to generate the initial estimate for the mapping function **u**, which depends on the focus-to-sample distance (*z*_1_) and any estimated degree of astigmatism in the lens system (which can occur when separate MLLs focusing in each orthogonal direction are not adjusted into a confocal condition and which would give rise to different magnifications of the projection images in those directions). This step is performed by the generate_pixel_map function, as shown in the top right of Fig. 2 ▸. We refer to the discrete representation of the mapping function as the ‘pixel mapping’.

Naturally, each image is discretely sampled by the pixel array of the detector. For pixel *i*, *j* of image *n*, the discrete representation of the image is given by *I*[*n*, *i*, *j*] ≡ *I*_*n*_(*i*δ*x*, *j*δ*y*), where δ*x* and δ*y* are the extent of a pixel along *x* and *y*, respectively, and we have used square brackets to indicate discrete arguments; here, we ignore the fact that pixels do not provide point samples of the diffraction but rather integrate its intensity over the domain of the pixel response function. Because the reference image is not measured by the detector, the sampling of *I*_ref_ is not constrained by the physical pixel geometry. Typically, we set the sampling period to equal the demagnified pixel size, so that *I*_ref_[*i*, *j*] ≡ *I*_ref_(*i*δ*u*, *j*δ*v*), where δ*u* = δ*x*/*M* and δ*v* = δ*y*/*M*. Translating the sample by Δ**x**_*n*_ in the transverse plane leads to a corresponding translation of the reference, because the convolution integral in the Fresnel propagator possesses translational equivariance. The sample translations are likewise converted to pixel units on the same grid, so that Δ*i*[*n*] = Δ*x*_*n*_/δ*u* and Δ*j*[*n*] = Δ*y*_*n*_/δ*v*, where Δ**x**_*n*_ = (Δ*x_n_* Δ*y_n_*). The function **u**(**x**) maps intensities from the detector grid to the reference image. Therefore, the sampling of the discrete representation of **u** is set by the detector geometry, and its values (coordinates in the reference image) are scaled by the chosen values of δ*u* and δ*v*. This leads to the discrete representation of the mapping function: *u*[0, *i*, *j*] ≡ (1/δ*u*)*u_x_*(*i*δ*x*, *j*δ*y*) and *u*[1, *i*, *j*] ≡ (1/δ*v*)*u_y_*(*i*δ*x*, *j*δ*y*), where **u**(**x**) ≡ [*u*_*x*_(**x**), *u*_*y*_(**x**)], and we make a similar definition for the discrete mapping **u**[*i*, *j*] ≡ (*u*[0, *i*, *j*], *u*[1, *i*, *j*]). Equation (1) can now be expressed in terms of these discrete quantities:
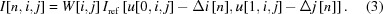



As part of the software suite, there are a number of helper functions for estimating the data sets necessary for initializing the main reconstruction loop. The function make_mask attempts to locate and mask bad pixels automatically. This is achieved by searching for pixels whose values deviate significantly from the median value of their neighbours during the scan. The output of this function can be viewed and manually modified by the mask_maker GUI. The make_whitefield function generates an estimate for the ‘white-field’ image by taking the median value of a pixel over the scan (with the sample in the beam). See p. 4 of the paper by Morgan, Murray *et al.* (2020 ▸) for a discussion of this approach. The function guess_roi will attempt to estimate the rectangular region of interest of the detector by choosing the rectangular region containing most of the signal in the white-field image. This region should include what is sometimes referred to as the ‘bright-field’ or holographic region of the diffraction, where the white-field image differs appreciably from 0.

We provide two helper functions to estimate the focus-to-sample distance: fit_thon_rings and fit_defocus_registration. In fit_thon_rings the defocus and the degree of astigmatism are approximated by fitting a forward model to the Fourier power spectrum of the data. With sufficient source coherence and signal-to-noise ratio, Fresnel fringes may be observed in each of the projection images. These fringes produce a ring-like pattern in the Fourier power spectrum, the ‘Thon’ rings (Thon, 1966 ▸; Spence, 2013 ▸): see for example Fig. 9 of Morgan, Murray *et al.* (2020 ▸). The spacing between these rings depends on the focus-to-sample distance along each of the transverse directions (see https://speckle-tracking.readthedocs.io/en/latest/thon_rings.html for details). This approach has the advantage that it is independent of the registered sample translations.

In the other approach, fit_defocus_registration, a reference image of the sample is built for a range of magnification factors. The average magnification factors are given by *M_x,y_* = 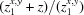, where 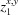 is the distance between the beam waist and the sample along the *x* or *y* axis, respectively. If the estimated magnification factors are far from the true values, then each of the projection images will be misregistered when forming the reference image and the resulting image will lack contrast. The values for 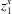 and 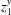 that produce the greatest image contrast are then chosen. This approach will probably fail if the initial estimates for the sample translation vectors deviate significantly from the true values. The aim of these two functions is to produce an estimate for the defocus that is sufficiently accurate for the subsequent iterative refinement process to converge, after which more accurate estimates for the defocus values can be obtained from the recovered phase profile.

### 2.2. Main loop   

Having formed an initial estimate for the pixel mapping function, the iterative reconstruction cycle typically consists of (i) generating the reference image; (ii) updating the pixel mapping between each image and the reference; (iii) updating the sample translation vectors; and (iv) calculating figures of merit. These four steps are described in the sections below.

**Figure 4:**
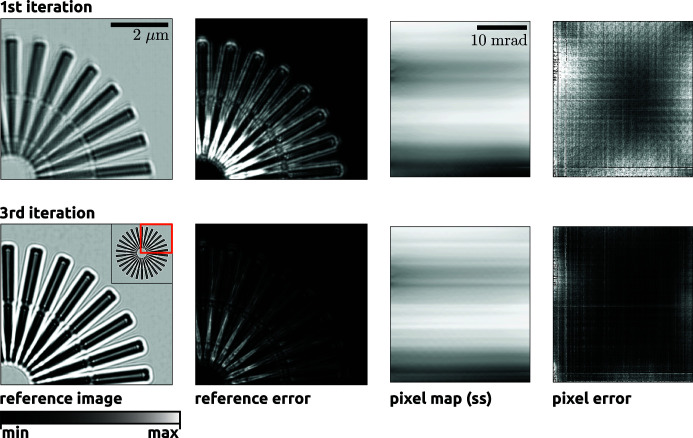
The reference image (first column) and the pixel map array (third column) along the detector’s slow scan axis (ss) (which corresponds to the vertical axis in this figure) for the first and third iterations of the ‘main loop’. In the first two columns we display only a subregion of the full field of view of the reference image, as indicated in the bottom left image by the orange outline in the top right corner. In the second and fourth columns we display the errors projected onto the reference image plane and the detector pixels, respectively (see Section 2.2.4). Each image is displayed on a linear greyscale colour map, with (min, max) values of (0.5, 1.3), (0, 3), (−5, 10) and (0, 400) for columns 1–4, respectively.

This approach is an *ad hoc* solution for minimizing the error function [equation (2)]. As such, convergence to the global solution is not guaranteed. Nevertheless, we have found that this simple approach performs well in many cases. For example, in Fig. 4 ▸ we display the reference image and pixel map after the first and third iterations of the above procedure. These data were recorded on the HXN beamline at the NSLS-II (Brookhaven National Laboratory, Upton, New York, USA) with a Siemens star test sample as the wavefront analyser object [see Morgan, Murray *et al.* (2020 ▸) for details]. The total error after the first loop was equal to 2.7 × 10^7^, after the third loop the error was reduced by a factor of three to 8.5 × 10^6^ and after ten iterations the algorithm converged to an error of 5.9 × 10^6^. See Section 2.2.4 for details of the error calculation.

#### 2.2.1. Make reference image   

The equation for updating the reference image is described in equation (27) of the paper by Morgan, Quiney *et al.* (2020 ▸). We present the discrete representation of this equation as an operation in pseudo code:

initialize *I*_ref_ and *w* (an array of the same size) with zeros

loop over all images (index *n*)

loop over all pixels (indexes *i* and *j*)

add the mapped intensities, weighted by *W*, to *I*_ref_:

*I*_ref_[*u*[0, *i*, *j*] − Δ*i_n_*, *u*[1, *i*, *j*] − Δ*j_n_*]] += *M*[*i*, *j*]*W*[*i*, *j*]*I*[*n*, *i*,*j*]

do the same for *W* ^2^, but add them to *w*:

*w*[*u*[0, *i*, *j*] − Δ*i_n_*, *u*[1, *i*, *j*] − Δ*j_n_*]] += *M*[*i*, *j*]*W* ^2^[*i*, *j*]

normalize the reference image by *w* for all [*i*, *j*]:

*I*_ref_ = *I*_ref_/*w*

where the symbols ‘+=’ signify the operation ‘add the value on the right to the value on the left’ and *M* is the pixel mask array, which is 1 for good pixels and 0 for bad. In cases where the values of *u*[0, *i*, *j*] − Δ*i*_*n*_ and *u*[1, *i*, *j*] − Δ*j*_*n*_ are non-integer in the pseudo code above, we use a sub-pixel interpolation régime for assigning these values to *I*_ref_ (see Appendix *A* [App appa] for details).

In the top left panel of Fig. 4 ▸, we show the reference image corresponding to the initial estimate for the pixel map as generated by generate_pixel_map. Looking closely, one can see that errors in the pixel mapping have resulted in placement errors, so that a given feature is misregistered when mapped to the reference image plane. After the third iteration, there are no visible placement errors and the Fresnel fringes near the edges of the Siemens star structure are now clearly visible.

#### 2.2.2. Update pixel mapping   

The pixel mapping is updated by minimizing the error function in equation (2) as a function of **u**. This minimization is performed in a brute-force search algorithm, where the error function is evaluated for every (typically) integer value of **u** within a predefined search window centred on the current estimate. We present the algorithm for updating the pixel mapping at pixel [*i*, *j*], based on equation (28) in the paper by Morgan, Quiney *et al.* (2020 ▸), in pseudo code:

initialize the error and variance (var) to zero

loop over search window along first axis (index *i*′)

loop over search window along second axis (index *j*′)

loop over all images (index *n*)

add the error for image *n* to error:

error[*i*′, *j*′] += *M*[*i*, *j*](*I*[*n*, *i*, *j*] − *W*[*i*, *j*]*I*_ref_[*i*′ − Δ*i_n_*, *j*′ − Δ*j_n_*])^2^

calculate variance:

var[*i*′, *j*′] += *M*[*i*, *j*](*I*[*n*, *i*, *j*] − *W*[*i*, *j*])^2^

normalize errors after all loops:

error = error/var

choose smallest normalized error:

*u*[0, *i*, *j*] = *i*′ and *u*[1, *i*,*j*] = *j*′ for [*i*′, *j*′] = argmin(error)

The size of the search window is selected by the user and should be large enough to accommodate the range of pixel shifts generated by the lens aberrations, but not so large as to make the calculation time impractical. The calculation time depends quadratically on the linear search window size.

This update procedure is performed over the discrete pixel grid, *i.e.* for integer values of *i*′ and *j*′. To obtain sub-pixel resolution, one can evaluate the error for non-integer values of *i*′ and *j*′, as described in Appendix *A* [App appa], or set the quadratic_refinement option to True in the update_pixel_map function. This quadratic refinement procedure was suggested by Zanette *et al.* (2014 ▸) and works by fitting a 2D paraboloid to the error profile in a 3 × 3 pixel window about the minimum value of the error to obtain sub-pixel precision.

This method, a grid-search algorithm with quadratic post-refinement, was chosen over gradient-based search algorithms after much trial and error. Upon examination of the error space, which is a 2D function for each pixel coordinate of **u**, we have often observed many local minima surrounding the global minimum. Such an error landscape can be problematic in gradient search algorithms, due to their tendency to converge to one of these local minima, and we speculate that this is why gradient-based minimization strategies have not worked well for our past experiments.

Since the mapping function is defined in terms of the gradient of a scalar function, **u** = **x** − λ*z*/(2π)∇Φ(**x**), the curl of the mapping function will be zero, ∇ × **u**(**x**) = 0, which applies in cases where Φ is non-singular and continuous. In such cases the vector field **u** is said to be ‘irrotational’. In the above procedure, however, the vector components of **u**, *u*[0, *i*, *j*] and *u*[1, *i*, *j*], were refined independently of each other. One can ensure that the mapping function is irrotational by first integrating **u** and then numerically evaluating the gradient to obtain an updated estimate for **u** with a curl of zero. The algorithm for integrating the pixel mapping is based on a least-squares conjugate gradient approach, also outlined by Zanette *et al.* (2014 ▸), and can be enforced by setting integrate to True in the call to update_pixel_map.

Finally, the pixel map can be filtered by setting the ‘sigma’ parameter in the call to the update function. This step serves to regularize the pixel mapping by filtering the pixel map with a Gaussian kernel, which can be useful in the early stages of the reconstruction to avoid artefacts that may arise from poor initial estimates of the reference image and sample translations.

In the third column of Fig. 4 ▸, we show the first component of the pixel map, *u*[0, *i*, *j*]. In the top row, the pixel mapping was generated using the above procedure, with the irrotational constraint applied and with a sigma value of 5 pixels, with the reference image shown in the first column. At the third iteration the sigma value was set to 1 pixel and thus finer details can be observed in the resulting map. Here, we have shown the pixel map after subtracting the linear term that arises from the estimated overall quadratic curvature of the phase.

In our example of Fig. 4 ▸, a pair of MLLs was used to focus the beam (Bajt *et al.*, 2018 ▸). Each MLL focuses the beam in one direction, like a cylindrical lens. We therefore expect that the dominant contribution to the lens aberrations would arise from placement and thickness errors in each layer of each of the 1D lenses. The orientation of each lens was aligned along the pixel axes of the detector [*i*, *j*], which also happen to be aligned to the *x* and *y* axes in the laboratory frame. Thus, assuming that each layer has a constant thickness in the direction perpendicular to the focusing axis, we would expect each component of the pixel map to vary along one axis only. That is, *u*_*x*_(**x**) would vary along the *x* axis and *u*_*y*_(**x**) would vary along the *y* axis. One can see in the pixel maps of Fig. 4 ▸ that this is indeed the case for *u*_*y*_(**x**). Such a property would not be expected, for example, in an axially symmetric lens, such as a compound refractive lens system.

#### 2.2.3. Update translations   

The procedure for updating the sample translation vectors follows the same logic as that for the pixel mapping update, as described in equation (29) of Morgan, Quiney *et al.* (2020 ▸). The error function of equation (2) can be evaluated as a function of Δ**x**_*n*_ by performing the integral over **x** but not the sum over *n*. For a given image, the error is then evaluated for integer pixel shifts of that image within a predefined search window. Once again, sub-pixel precision is achieved by fitting a 2D paraboloid to the resulting error terms in a 3 × 3 pixel window about the minimum value. This process is then repeated for every image.

#### 2.2.4. Calculate figures of merit   

The normalized error term in the integrand of equation (2) yields the error at a given pixel for a given image, ∊[*n*, *i*, *j*]. The function calc_error performs this calculation and then integrates the resulting errors: (i) over every image, yielding the ‘pixel error’ ∊[*i*, *j*]; (ii) over every pixel, yielding the ‘frame error’ ∊[*n*]; and (iii) over every pixel and image, yielding the ‘total error’ ∊. Following the same procedure used to form the reference image, one can also map the errors at each pixel for each image to the reference image plane, yielding the ‘reference image error’. By projecting the error onto these different bases, it is easier to estimate the origin of potential artefacts in the reconstruction.

For example, in the second and fourth columns of Fig. 4 ▸, we show maps of the reference image error and the pixel error, respectively. In the reference image error we can see that errors are localized near the centre of the Siemens star projection image, where the Fresnel fringes from neighbouring edges begin to overlap.

### 2.3. Additional analysis   

The function calculate_phase generates the phase profile of the wavefront produced by the focusing optics (based on the recovered pixel map), focus_profile generates the profile of wavefront intensities near the focal plane and zernike calculates the aberration coefficients from the recovered phase profile.

The function split_half_recon takes every pixel of every image in the input data set and randomly assigns it to one of two separate data sets. Two pixel maps are then generated by comparing the existing reference image with each of the halved data sets. A histogram of the differences between these reconstructed pixel maps then provides an estimate for the underlying uncertainty in the original reconstruction.

The function calculate_sample_thickness is an implementation of ‘Paganin’s algorithm’, where the thickness profile can be reconstructed from the reference image [see, for example, equation (61) in the paper by Paganin & Pelliccia (2019 ▸), which is based on a low-order expansion of the transport of intensity equations (TIE) (Teague, 1983 ▸)]. This function is useful in that it provides a quick estimate for the sample’s thickness profile (as opposed to the reference image which merely provides a projected in-line hologram of the sample) but is likely to be inaccurate for many applications. Other, more advanced, tools exist for this purpose; see for example, the *X-TRACT* software package by Gureyev *et al.* (2011 ▸).

## 3. The coherent X-ray imaging file format   

**Figure 5:**
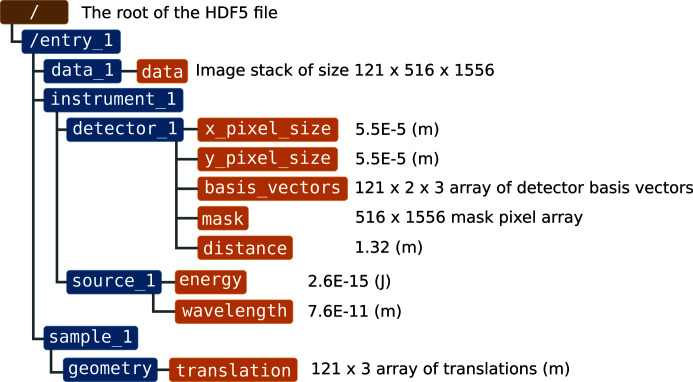
A diagram of a CXI file containing 121 images recorded on a 516 × 1556 pixel array detector.

The *speckle-tracking* software suite uses the coherent X-ray imaging (CXI) file format (Maia, 2012 ▸) for input and output of key parameters and data sets. The CXI format is based on the popular HDF5 format, which is a self-describing container for multidimensional data structures. The CXI format can be understood as simply a set of conventions for storing scientific data relating to coherent X-ray imaging in an HDF5 file. For example, the simplest input file for the *speckle-tracking* program has the structure illustrated in Fig. 5 ▸.

The CXI file format solves a number of common problems when translating data between programs and facilities: it establishes a default unit system (SI units), a default coordinate system, and a means of describing the relative orientation of the detector modules with respect to the laboratory frame of reference (via the basis_vectors). Providing a self-describing and unambiguous container for scientific data is also the aim of the NEXUS file format (Maddison *et al.*, 1997 ▸), which is likewise built on top of the HDF5 format. However, perhaps due to NEXUS’s generality and scope, NEXUS-formatted files can be extremely complex, such that it can become difficult to extract meaningful data without specialized software. Thus, the CXI format was chosen as a suitable compromise between the aims of simplicity and an unambiguous representation of the data.

The output of the various programs within the *speckle-tracking* suite can be stored in the same CXI file as the input, by default within the group called /speckle_tracking, or in another HDF5 file entirely, depending on the user configuration. Thus, the program’s output data may, or may not, follow the default classes listed in the CXI standard.

### 3.1. Coordinate system   

**Figure 6:**
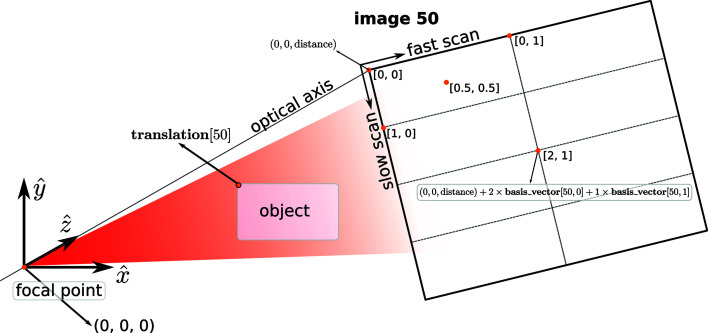
A diagram showing the relative positions of the focal point, the object and the detector pixels in terms of quantities stored in the CXI file.

One point of difference between the CXI standard and that adopted by the *speckle-tracking* software relates to the default origin of the coordinate system. In the CXI standard, the origin of the laboratory frame is centred on the sample of interest, with 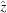 parallel to the beam axis and 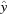 pointing against the direction of gravity in a right-handed coordinate system. In many of our experiments, the sample’s *z* position is not known accurately in advance and often changes during the course of the experiment. Therefore, we have set the focal point of the optical system as the origin of the coordinate system, so that estimates of the sample’s position may be refined *post facto* without needing to offset the coordinates for the detector array and the focal point. Otherwise, we have followed the CXI standard as shown in Fig. 6 ▸.

By default, the [0, 0] pixel of the detector array is assumed to be centred on the optical axis with *z* = distance (located in the /entry_1/instrument_1/detector_1 group of the CXI file). If this is not the case, then another data set can be added to specify the corner position of the [0, 0] pixel for each image. Currently, no part of the software suite actually makes use of such additional information, since the effect of an unknown translation (in the transverse plane) relative to the optical axis is to induce a corresponding offset in the reference image reconstruction in addition to a linear phase gradient on the wavefront reconstruction, which in any case is typically removed in the post-reconstruction analysis.

The (*x*, *y*, *z*) coordinate of each pixel with respect to the pixel origin, *i.e.* pixel [0, 0] at (0, 0, distance), is defined by the basis_vector data set. This is a 3D array, where the first dimension corresponds to the image number, the second to the detector axes and the third to the three spatial dimensions. These vectors encode both the direction and magnitude of one step along the fast or slow scan axes of the detector array. Thus, the (*x*, *y*, *z*) location of pixel [2, 1] in image 50 can be obtained by starting at the location of pixel [0, 0] at (0, 0, distance) and stepping twice along the slow scan axes of the detector, followed by one step along the fast scan axis: (0 0, distance) + 2 × basis_vector[50, 0] + 1 × basis_vector[50, 1].

## 4. Software availability   

In order to maximize the utility and accessibility of the software suite, *speckle-tracking* is available under Version 3 or later of the GNU General Public Licence. This allows for other software projects to incorporate and modify all or part of this program into their own processing pipeline. The software can be downloaded from https://github.com/andyofmelbourne/speckle-tracking.

## 5. Documentation   

High-level documentation for the project, including tutorials and installation instructions, can be found at https://speckle-tracking.readthedocs.io. For help with the command-line programs, simply pass --help as an argument when calling the program. This will provide a brief description of the program and the list of parameters accepted in the configuration file. Similar descriptions are provided in the ‘doc string’ of the Python functions. In each of the GUIs, descriptions of the input parameters can by viewed in a pop-up window, which is displayed when hovering the mouse over the parameter name.

## 6. Available speckle-tracking data sets   

Currently, there are three experimental data sets available to download at the Coherent X-ray Imaging Data Bank (https://www.cxidb.org), data sets 134–136. Each data set is stored in the CXI file format, which can be used immediately as input for the *speckle-tracking* software suite. For testing with other programs, individual data sets and parameters within the file can be extracted and converted to other formats using one of the many HDF5 APIs (available at https://www.hdfgroup.org). Tutorials (found at https://speckle-tracking.readthedocs.io/en/latest/#tutorials) have been written for two of the available data sets, which is the recommended way of reproducing the results in this article and becoming familiar with the software suite.

## Figures and Tables

**Figure 7:**
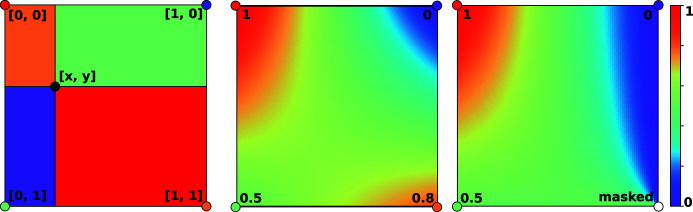
(Left) A diagram showing the relative weights corresponding to the values at each corner of the unit square. The weights corresponding to each point are equal to the area occupied by the rectangle diagonally opposite and are colour coded to illustrate this. (Middle) The interpolated values for all points within the unit square, given the values shown at each vertex. (Right) The same as the middle panel, but for the case where the value at the bottom right corner is unknown.

## Footnotes

2 Note that for near-field ptychography a diffuser is often employed to add structure to the illumination and improve robustness. Nevertheless, near-field ptychography is distinct from XST techniques.

## Appendix A Sub-pixel interpolation

In the *speckle-tracking* software suite, it is frequently the case that (i) the value of an array must be evaluated at fractional pixel coordinates or (ii) a value must be added to an array at a location with fractional pixel coordinates. After experimenting with a few common solutions to this problem, we have elected to use the bilinear interpolation formulation (Press *et al.*, 1988 ▸). This is a simple extension of 1D linear interpolation to the problem of interpolating a function of two variables.

If the values of a 2D array represent discrete samples of a continuous function over a Cartesian grid, with a fixed and equal pixel spacing along both dimensions, then bilinear interpolation reduces to a simple form. Consider the four values of some array, *f*[0, 0], *f*[1, 0], *f*[0, 1] and *f*[1, 1], which are point samples of the continuous function *f*(*x*, *y*) at each corner of a unit square. The value of *f* at some point [*x*, *y*] that is within this unit square can be approximated by

This corresponds to subdividing the unit square into four rectangular segments, centred on the point [*x*, *y*], so that the weighting given to a particular corner value corresponds to the area occupied by the rectangle diagonally opposite its position, as shown in the left panel of Fig. 7 ▸. Since the total area of the four rectangles is equal to that of the square, the four weighting factors sum to 1. However, if one of the corner values is unknown, then this value can be excluded from the interpolation routine and the remaining values can be re­normalized. Setting *f*_*M*_ ≡ *f* × *M*, where *M* is a mask array consisting of ones (for good values of *f*) and zeros (for bad or missing values of *f*), then the values of *f*(*x*, *y*) are approximated by

In Fig. 7 ▸ (right panel), we show an example of the distribution of values when the bottom right corner value is unknown, for comparison with the case in the central panel where all four corner values of *f*(*x*, *y*) are known.

Conversely, when assigning a given value of *f* at the fractional coordinate [*x*, *y*] to the discrete array at each of the corner positions, the same weighting scheme is employed. If there exists more than one value of *f*(*x*, *y*) to interpolate onto the discrete grid, then the weighting factors are stored in an array for renormalization after all points have been assigned.

One disadvantage of this approach is that it is not invertable. For example, interpolating an array of values onto a regular grid that is translated with respect to the original grid by some fraction of a pixel, followed by a corresponding shift in the opposite direction, does not preserve the original values of the array. Furthermore, interpolated values can have a discontinuous gradient when crossing the boundary between pixels, even when the underlying function *f*(*x*, *y*) has continuous first derivatives.

At the same time, however, this approach has a number of advantages. For example, sharp edges or points of high contrast in the original array do not produce artefacts that span the entire domain of the interpolated array, which can arise when using Fourier-based interpolation routines. Because the interpolation is local, only pixel values in the immediate neighbourhood of a point [*x*, *y*] are needed to compute the interpolated value. This property is particularly advantageous for parallel computations. In addition, masked values can be easily accommodated by renormalizing each of the weighting terms.
